# The association between fear of falling and occurrence of falls: a one-year cohort study

**DOI:** 10.1186/s12877-022-03018-2

**Published:** 2022-05-05

**Authors:** Tsuyoshi Asai, Kensuke Oshima, Yoshihiro Fukumoto, Yuri Yonezawa, Asuka Matsuo, Shogo Misu

**Affiliations:** 1 Department of Physical Therapy, Faculty of Rehabilitation, Kobe Gakuin University, 518 Ikawadanicho, Arise, Nishi-ku, Kobe, Hyogo 651-2180 Japan; 2 Faculty of Rehabilitation, Kansai Medical University, 18-89 Uyamahigashicho, Hirakata, Osaka 573-1136 Japan; 3 Everehab, Inc., 46 Kamitakanonakamachi, Sakyo-ku, Kyoto-city, Kyoto, 606-0044 Japan; 4 Inami Town Office, 1-1 Kunioka, Inami town, Kako-gun, Hyogo, 675-1115 Japan; 5 Inami-Cho Social Welfare Council, 4369-3 Kako, Inami town, Kako-gun, Hyogo, 675-1105 Japan; 6 Department of Physical Therapy, Faculty of Nursing and Rehabilitation, Konan Women’s University, 2-23, 6 Chome, Morikita-machi, Higashinada-ku, Kobe, Hyogo, 658-0001 Japan

**Keywords:** Fear of falling, Fall history, Fall occurrence, Community-dwelling older adults

## Abstract

**Background:**

Both multiple fall experiences and fear of falling (FoF) would make people susceptible to another fall; however, the associations are unknown. This study investigates the association of FoF with fall occurrence among older adults according to their fall history.

**Methods:**

In this study, we adopted a longitudinal observational design. We visited 20 community centers to recruit 1,025 older adults (aged 65 years or older). At baseline, FoF was assessed using a single-item questionnaire. The number of falls in the past year was obtained via a self-questionnaire and participants were classified into three fall history groups (0: non-faller, 1: single faller, 2 or more: multiple faller). After a year of following-up, the number of falls during the year was considered as the main outcome. Poisson regression models clarified the influence of FoF on fall occurrence during the one-year follow-up, according to the participants’ fall history.

**Results:**

The final sample comprised 530 individuals (follow-up rate: 530/801, 66.4%). Fall history, FoF, and interaction between multiple fallers and FoF were significant in the adjusted statistical model (rate ratio [95% confidence interval]: single faller = 2.81 [1.06, 6.30], multiple faller = 13.60 [8.00, 23.04], FoF = 3.70 [2.48, 5.67], multiple faller*FoF = 0.37 [0.20, 0.68]).

**Conclusions:**

We found that FoF was associated with the occurrence of falls in community-dwelling older adults. However, its association was lower in multiple fallers.

## Introduction

Fear of falling (FoF) is an emotional feeling among older adults. It is defined as “a person's anxiety towards usual or normal walking or mobilizing, with the perception that a fall will occur” [1]. Some studies have shown that FoF influences walking, and suggest that FoF indirectly affects the occurrence of falls [2–4]. Supporting this research, FoF is considered a significant predictor of occurrence of falls in community-dwelling older adults [5, 6]. A systematic review has shown that individuals with FoF have more than double the risk of falling than those without FoF [6]. Additionally, some studies reveal that FoF is associated with various changes, which increase the risk of falls (e.g., inappropriate attention allocation, fixed vision trajectory, and an inactive lifestyle) [7–9]. Considering the strong association between FoF and occurrence of falls, it is important for health-related workers in the community to obtain FoF information. The development of effective prevention programs for falls has been a long-term issue in geriatric research, and a better understanding of the influence of FoF on the occurrence of falls is considered important [10].

Fall history is another strong predictor of future falls, affecting the development of FoF in older adults [4, 5]. Importantly, fall history has different meanings according to the number of falls (zero, one, two or more) [11–19]. Studies examining multiple fallers separately from non- and single fallers have pointed out significant differences between them regarding the influence of health outcomes [12, 13, 15]. Taking physical function as an example, multiple fallers showed significantly lowered gait ability and balance functions than others [11–15, 17–19]. The effect of FoF on fall occurrence in the future could also vary according to fall history (number of falls: zero, one, two or more). Moreover, coupling the characteristics of multiple fallers and FoF would affect fall occurrence; however, observational studies regarding this effect have not been conducted.

In the present study, we investigated the association of fall occurrence with fall history and FoF, including the interaction between these two factors in statistical models. We hypothesized that FoF is associated with the fall occurrence; however, its association is not consistent with fall history. Therefore, this study aimed to clarify this association, hinting at the development of assessment tools and a new clinical approach for preventing falls.

## Methods

### Participants

The present study adopted a longitudinal observational design. We visited 20 community centers for older adults in Inami town, Hyogo prefecture, Japan, from April to October 2016 and recruited 1,025 individuals. Participants who met the following criteria were included: (1) aged 65 years or older, and (2) ability to walk independently with/without an assistive device. Those with (1) cognitive impairment (rapid dementia screening test score < 8) [20], (2) self-reported neurological disease (stroke and Parkinson's disease), and (3) missing data were excluded. The study was conducted in accordance with the principles of the Declaration of Helsinki. The Research Ethics Committee of Kobe Gakuin University approved this study (approval no. HEB100806-1). Informed consent was obtained from all participants prior to participation.

### Data collection

At the baseline, demographic characteristics of participants were collected using a self-administered questionnaire. The questionnaire items included age, sex, height, weight, presence of neurological disease (stroke and Parkinson's disease), number of drugs taken per day, number of falls in the past year, and FoF. A fall was defined as “an event that resulted in the participant unintentionally coming to the ground or another lower level” [21]. The FoF was assessed using the question “Are you afraid of falling (yes/no)?” This format has been reported to have a high test–retest reliability and has the advantage of being straightforward. It also allows for the easy generation of prevalence estimates [22–24]. To assess physical mobility, timed up and go test (TUG) was conducted [25]. In the TUG, participants were asked to stand up from a seated position in a standard armchair, walk 3 m, turn around, walk back to the chair, and sit down again. The time taken to complete the test was measured using a digital stopwatch [25]. Participants wore their usual footwear and walked at a comfortable and safe pace. At the one-year follow-up measurement, the number of falls in the past year (main outcome), obtained using a self-administered questionnaire was considered the main outcome in this study.

### Statistical analysis

Based on the baseline information on the number of falls in the past year, the participants were classified into three fall history groups: non-fallers (number of falls: 0), single fallers (number of falls: 1), and multiple fallers (number of falls: > 2). The dummy variables were age (0: 65–69 years, 1: 70–75 years, 2: 75–79 years, 3: 80–84 years, 4: 85 years or older), FoF (0: no, 1: yes), sex (0: male, 1: female), TUG test (0: < 13.5 s, 1: ≧ 13.5 s) [26], polypharmacy (0: < 4, 1: ≧ 4) [27], and fall history group (0: non-fallers, 1: single fallers, 2: multiple fallers). We used descriptive statistics and chi-square tests to describe the prevalence of baseline characteristics according to fall history.

Poisson regression models were used to clarify the influence of FoF on the number of falls according to fall history during the one-year follow-up, yielding rate ratios (RRs) and 95% confidence interval (CI). Model 1 included FoF, fall history group, and fall history group*FoF (as an interaction) as explanatory variables. Model 2 included all explanatory variables, with age, sex, TUG test, and polypharmacy as confounding variables. To examine similarities between individuals included in the final sample and those who dropped out, group comparisons were conducted for the variables. The significance level was set at *p* < 0.05. All statistical analyses were conducted using R ver. 4.14 (R information).

## Results

**Fig. 1:**
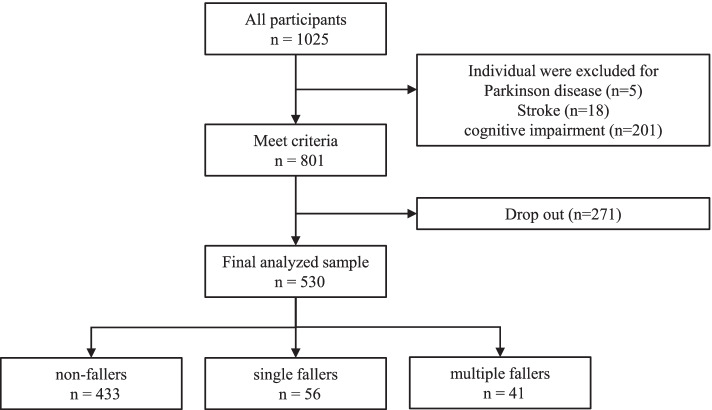
Flow chart of study sample

A flow chart of the study sample is shown in Fig. 1. Of the 1,025 participants, 236 did not meet the inclusion criteria (Parkinson’s disease = 5, stroke = 18, cognitive impairment = 201, missing data = 12). The final baseline sample comprised 789 participants. At the one-year follow-up, 259 participants dropped out. The final analytical sample consisted of 530 individuals (follow-up rate: 530/801, 66%).

Based on fall history, 433, 56, and 41 individuals were classified as non-fallers, single fallers, and multiple fallers, respectively. The baseline characteristics of the participants are presented in Table 1. The prevalence of FoF in single and multiple fallers was higher than in non-fallers (non-fallers, 217/433 (50.1%); single fallers, 40/56 (71.4%); multiple fallers, 29/41 (70.7%); *p* = 0.001). Multiple fallers exhibited decreased physical functionality (slower TUG) than non- and single fallers (non-faller, 5/433 (1.2%); single fallers, 1/56 (1.8%); multiple fallers, 3/41 (7.3%); *p* = 0.014). The ratio of individuals with polypharmacy increased with an increase in fall history (non-faller, 65/433 (15.0%); single fallers, 13/56 (23.2%); multiple fallers, 17/41 (41.5%); *p* < 0.001). Regarding baseline characteristics, no significant differences existed between the dropouts and the participants in the final analytical sample (Table 2).

**Table 1:** Characteristics of participants at baseline

	Non-fallers (*n*, % = 433, 81.7)	Single fallers (*n*, % = 56, 10.6)	Multiple fallers (*n*, % = 41, 7.7)	*p*-value
Age, n (%)				
65–69	57 (13.2)	5 (8.9)	2 (4.9)	0.095
70–74	156 (36.0)	21 (37.5)	10 (24.4)	
75–79	123 (28.4)	23 (41.1)	14 (34.1)	
80–84	70 (16.2)	6 (10.7)	11 (26.8)	
Above 85	27 (6.2)	1 (1.8)	4 (9.8)	
Fear of fall, n (%)	217 (50.1)	40 (71.4)	29 (70.7)	0.001
Female, n (%)	286 (66.1)	41 (73.2)	27 (65.9)	0.559
Slower TUG, n (%)	5 (1.2)	1 (1.8)	3 (7.3)	0.014
Polypharmacy, n (%)	65 (15.0)	13 (23.2)	17 (41.5)	< 0.001

*TUG* timed up and go test

Participants whose TUG score was less than 13.5 s were classified as slower in TUG

**Table 2:** Group comparison between individuals in analytical sample and drop-out individuals

	Analytical sample (*n* = 530)	Drop out (*n* = 217)	*p*-value
Age, n (%)			
65–69	64 (12.1)	51 (18.8)	0.106
70–74	187 (35.3)	93 (34.3)	
75–79	160 (30.2)	73 (26.9)	
80–84	87 (16.4)	36 (13.3)	
Above 85	32 ( 6.0)	18 (6.6)	
Fear of fall, n (%)	286 (54.0)	142 (52.4)	0.730
Female, n (%)	354 (66.8)	164 (60.5)	0.093
Slower TUG, n (%)	9 (1.7)	3 (1.1)	0.744
Polypharmacy, n (%)	95 (17.9)	45 (17.0)	0.818

*TUG* timed up and go test

Participants whose TUG score was less than 13.5 s were classified as slower

**Fig. 2:**
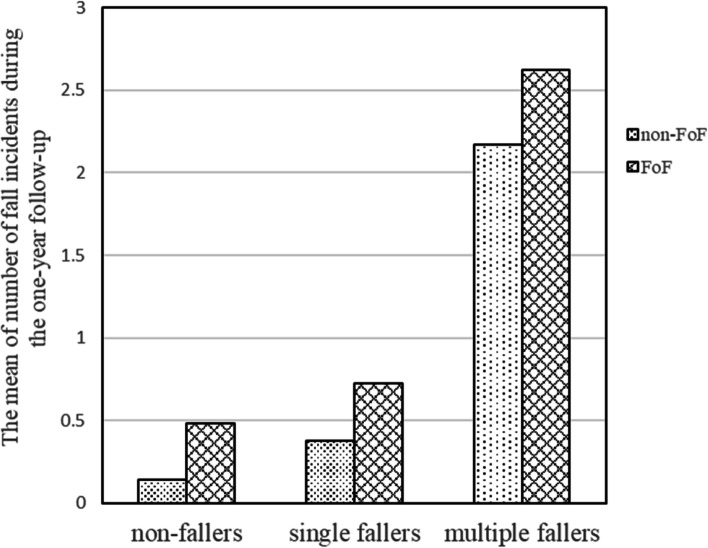
The mean of number of fall incidents during the one-year follow-up according to self-reported fall history and FoF at the baseline measurement FoF: fear of fall. Fall history groups: Based on the self-reported fall history at baseline, the participants were classified into non-fallers (number of falls: 0), single fallers (number of falls: 1), and multiple fallers (number of falls: > 2). FoF groups: Based on the assessment of fear of falling at baseline, the participants were classified into FoF and non-FoF

The mean of number of fall incidents during the follow-up year according to fall history classified by FoF is shown in Fig. 2 (non-faller without and with FoF, number of falls/person: 0.14 (31/216) and 0.48 (104/217), single faller without and with FoF: 0.38 (6/16) and 0.73 (29/40), multiple faller without and with FoF: 2.2 (26/12) and 2.6 (76/29). The results of Poisson regression models for the fall-related measures (fall history group, FoF, and interaction between fall history group and FoF) are shown in Table 3. Fall history group (single and multiple fallers); FoF; and interaction between multiple fallers and FoF, but not between single fallers and FoF; were found to be significant in Model 1 (RR [95% CI]: single fallers = 2.61 [0.98, 5.83], multiple fallers = 15.10 [8.90, 25.40], FoF = 3.34 [2.27, 5.07], multiple fallers*FoF = 0.36 [0.20, 0.66]). These parameters remained significant after adjusting for confounders in Model 2 (RR [95% CI]: single fallers = 2.81 [1.06, 6.30], multiple fallers = 13.60 [8.00, 23.04]; FoF = 3.70 [2.48, 5.67]; multiple fallers*FoF = 0.37 [0.20, 0.68]).

**Table 3:** Rate ratios for the occurrence of falls during one-year follow-up according to fall-related measures (fall history, FoF, fall history*FoF)

	Model 1		Model 2	
Variables	RR	95%CI	RR	95%CI
Fall history				
non-faller	Ref		Ref	
single fallers	2.61	0.98, 5.83	2.81	1.06, 6.30
multiple fallers	15.10	8.90, 25.40	13.60	8.00, 23.04
FoF				
non-FoF	Ref		Ref	
FoF	3.34	2.27, 5.07	3.70	2.48, 5.67
Interaction, fall history*FoF				
non-faller*FoF	Ref		Ref	
single fallers*FoF	0.58	0.23, 1.66	0.48	0.19, 1.38
multiple fallers*FoF	0.36	0.20, 0.66	0.37	0.20, 0.68

Model 1 did not include any confounding variables. Model 2 included age, sex, TUG, and polypharmacy as confounding variables

*FoF* fear of falling, *RR* rate ratio, *CI* confidence interval, *TUG* timed up and go test

## Discussion

In this longitudinal observational study, we investigated the association of FoF with fall occurrence according to fall history in the previous one year (non-fallers, single fallers, and multiple fallers) for community-dwelling older adults. Our results indicate that FoF was significantly associated with the occurrence of falls in community-dwelling older adults. However, the association was not consistent with fall history at baseline.

First, the results of Poisson regression models showed that the FoF was significantly associated with fall occurrences even after controlling for confounders, and the RR was 3.70. These results indicate that FoF is a significant risk factor affecting fall occurrence, and that the assessment of FoF is useful to predict fall occurrences in community-dwelling older adults. These results support previous research, showing FoF as a fall risk factor [5, 28]. A systematic review has shown that screening for FoF should be prioritized to detect high risk older adults, along with fall history [28]. For clinical staff members, the assessment of FoF is strongly recommended to find older adults who are prone to fall. Additionally, our results showed that the ratio of individuals with FoF increased with an increase in past fall experiences. Some studies have reported people developing FoF without fall experiences, and that FoF is not persistent but transient and can be overcome [29, 30]. This indicates that the experience of falling can lead to FoF; thus, preventing a fall is important to break this cycle.

Hereafter, our results showed that the RRs [95% CI] of interaction between FoF and single fallers and multiple fallers were 0.48 [0.19, 1.38] and 0.37 [0.2, 0.68], respectively. Notably, the RRs of the interactions were lower than one and lowered with increased fall history. These results indicate that the association of FoF with fall occurrence may vary with fall history, with multiple fallers showing lower association of FoF with fall occurrence than single fallers and non-fallers. To our knowledge, no study has investigated the association of FoF with the fall occurrence in multiple fallers. This study provides insights into FoF for clinicians and researchers in this field. The results for multiple fallers can be partially explained by their characteristics. In some studies, multiple fallers showed significantly lowered levels in specific physical functions such as vision and stepping movement when turning, which cannot be captured by general physical assessment [12, 13, 15]. Similarly, the results of systematic review suggest that the information on fall history includes psychological and behavior risks [5]. This implies that having a multiple fall history itself may be a strong predictor for falls compared to other factors. Additionally, FoF-associated physical activity restriction might be a crucial factor that helps in explaining our results [31–33]. The FoF-associated physical activity restriction tends to occur in multiple fallers [31]. It leads to significant lowered physical function, which may cause the person to suffer a fall again [32, 33]. In the present study, the measurement of physical activity was not included; thus, in the future, a study is needed that includes the same.

As mentioned above, the interaction between single fallers and FoF was not significant in Poisson regression models. This result indicates that one-time fall history does not affect the association of FoF with fall occurrence, that is, FoF possibly affect non-fallers and single fallers equally in terms of fall occurrences. This result can be partly explained by single fallers’ characteristics. Older adults with high physical functions may fall accidentally by exposing themselves to high fall risk situations [34]. Such people may be classified as single fallers in the present study and may show physical performance similar to non-fallers, which is supported by the slower TUG result. Additionally, although the interaction was not significant, a single fall was associated with fall occurrence and the RRs of single fallers were much smaller than those of multiple fallers. These results are reflective of that of other studies, suggesting that single fallers are an important fall risk group, but they are at lower risk than multiple fallers [16–18].

There are some limitations to this study. First, the dropout rate was over 30% (271/801, 34%) and selection bias may have occurred. Even though the results of characteristic comparison between individuals who dropped out and those in the final analytical sample did not differ significantly, potential confounders may still exist. Second, we obtained the number of falls in the past year at both baseline and one-year follow-up via a self-administered questionnaire; thus, recall bias may have occurred. Older adults with FoF possibly have a bias regarding their fall history. For example, those who had FoF may have more easily remembered the previous falls. This implies that FoF and fall history may have a mutual relation, which leads to misclassification of the fall history at baseline. This may affect our results. Third, FoF was measured using a one-item questionnaire. This method is an easy and quick way to assess the prevalence of FoF as mentioned in the introduction. However, the influence of FoF on occurrence of fall may vary according to the degree of FoF. Future studies should assess FoF using a one-item questionnaire, and a multiple-item questionnaire which can quantify FoF, such as fall efficacy scale [6]. Fourth, other risk factors such as mental health issues, eye problems, pain, etc. were not measured in our study [5, 35]. These factors may affect our results. Finally, there was a small sample size for multiple fallers; thus, the generalizability is not high. A well-designed study is warranted in the future to overcome the limitations of the present study.

In conclusion, FoF was associated with the occurrence of falls among community-dwelling older adults, and its association was not consistent with fall history at baseline. Compared with non-fallers, the association of FoF with fall occurrence was smaller in multiple fallers. Our results have implications for clinical staff members to interpret the association of FoF with fall occurrence.

## Data Availability

The data that support the findings of this study are available on request from the corresponding author [TA]. The data are not publicly available due to them containing information that could compromise research participant privacy/consent.

## Abbreviations

FoF: Fear of falling
TUG: Timed up and go test
RR: Rate ratio
CI: Confidence interval
